# Case Report: Sepsis With Multisystemic Amyloid Light‐Chain Amyloidosis

**DOI:** 10.1155/crcc/6794847

**Published:** 2026-10-26

**Authors:** Mehtap Selçuk, Galip Emre Kılıçaslan, Sinem Keskin Kayalar, Ismail Cinel

**Affiliations:** ^1^ Acıbadem Ataşehir Hospital Istanbul Turkey; ^2^ Department of Anesthesiology and Reanimation Department of Intensive Care Acıbadem University Medical Faculty Istanbul Turkey acibadem.edu.tr

**Keywords:** amyloid nephropathy, amyloid purpura, amyloidosis, sepsis, septic shock

## Abstract

**Introduction:**

Systemic light‐chain (AL) amyloidosis is a complex disorder characterized by extracellular fibril deposition, leading to severe multisystem dysfunction. When sepsis superimposes on preexisting organ impairment, early clinical recognition becomes particularly challenging yet critical.

**Methods:**

We present a case of a 60‐year‐old female who presented with generalized edema, dyspnea, and cardiac/renal involvement, subsequently diagnosed with AL amyloidosis based on laboratory, imaging, bone marrow biopsy, and Congo red staining findings.

**Results:**

Following chemotherapy with bortezomib and cyclophosphamide, the patient experienced transient cardiac arrest, received an implantable cardioverter defibrillator, and subsequently developed massive ascites. Upon developing *Pseudomonas guariconensis* bacteremia, she progressed to septic shock requiring intensive care unit admission, mechanical ventilation, vasopressor support, and targeted antimicrobial therapy. Through aggressive supportive care and source control, hemodynamic stabilization was achieved, allowing successful extubation and transfer to the general ward.

**Conclusions:**

Superimposed sepsis on severe, multi‐organ amyloidosis significantly complicates diagnosis and management. High clinical vigilance, early infection recognition, and prompt targeted therapy are essential to prevent fatal outcomes in patients with preexisting chronic organ dysfunctions.

## 1. Introduction

Systemic amyloidosis, as a chronic noninfectious inflammatory condition, comprises a heterogenous group of disorders characterized by the extracellular deposition of insoluble, misfolded protein fibrils (termed amyloid) in multiple systems. These fibrils adopt a beta‐plated sheet conformation, which confers biochemical stability but also pathogenic insolubility, leading to progressive organ dysfunctions [1]. The classification of systemic amyloidosis is based on the precursor protein, with the most clinically significant subtypes including immunoglobulin light chain (AL) amyloidosis [2]. The clinical presentation is highly variable and largely determined by the specific organs involved, such as the kidneys, heart, liver, peripheral nervous system, and gastrointestinal tract being affected. Definitive diagnosis requires histopathological confirmation via tissue biopsy demonstrating amyloid deposits [3].

During the hospitalization period related to organ dysfunctions, sepsis [3] represents an infectious inflammatory significant contributor to both morbidity and mortality. Its management primarily depends on infection control and supportive care for organ functions. To maximize the effectiveness of these interventions, they must be initiated promptly, underscoring the critical importance of healthcare professionals’ awareness for early sepsis recognition and diagnosis [4].

This case report aims to present a patient with systemic amyloidosis involving the renal, cardiac, and hepatic systems that superimposed sepsis and septic shock.

## 2. Case Presentation

As this is a retrospective case report without any experimental interventions, formal ethics committee approval is not required. However, written informed consent was obtained from the patient’s family for the publication of this case report and any accompanying clinical details and images.

A 60‐year‐old woman with no prior history of chronic illness, medication use, or allergies presented to an external center 5 months earlier with complaints of generalized edema, fatigue, dyspnea, and an unintentional weight loss of 8 kg over the preceding 2 months. Initial evaluation revealed prerenal azotemia, and whole‐body magnetic resonance imaging demonstrated findings suggestive of cardiac amyloidosis. The patient was subsequently referred to our center for further work‐up. Laboratory results demonstrated findings consistent with amyloidosis:

### 2.1. Laboratory Findings

• HbA1c: 6.9%
• Serum amyloid A: 2.73 mg/dL
• Spot urine protein/creatinine ratio: 0.16 mg/mg
• Serum protein electrophoresis (Figures 1 and 2):
  o. Total protein: 68 g/L
  o. Albumin: 54.8%
  o. Alpha‐1 globulin: 6.1%
  o. Alpha‐2 globulin: 8.9%
  o. Beta‐1 globulin: 7.7%
  o. Beta‐2 globulin: 6.1%
  o. Gamma globulin: 16.4%
• Serum *β*2‐microglobulin: 7.05 mg/L
• Spot urine *β*2‐microglobulin: 0.22 mg/L

**Figure 1:**
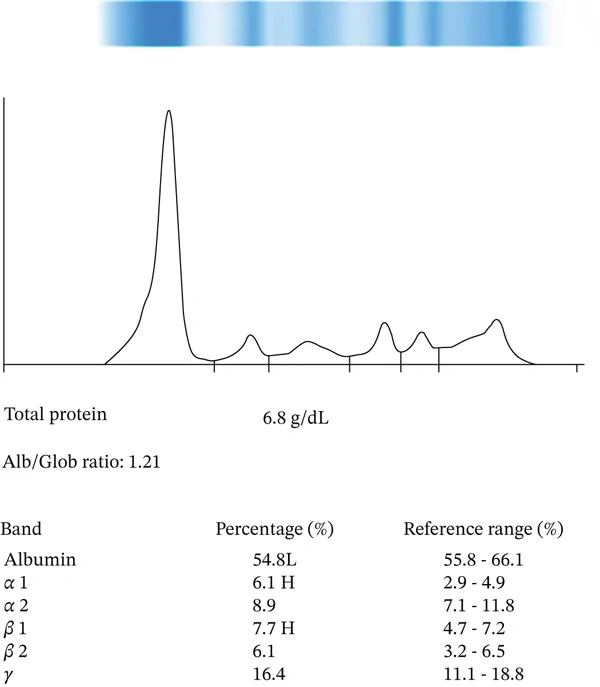
Serum protein electrophoresis.

**Figure 2:**
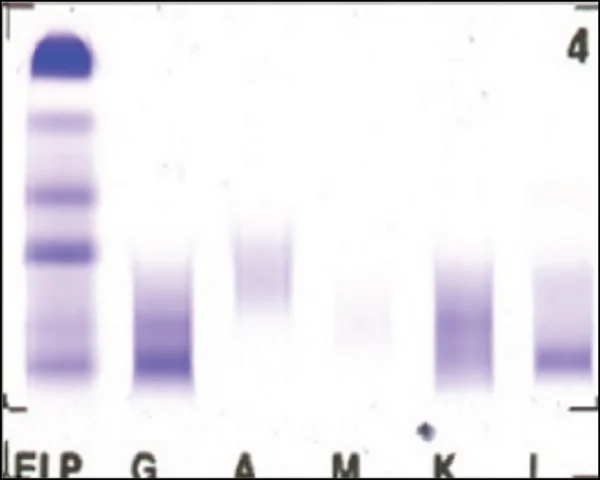
Serum immunofixation electrophoresis.

Bone marrow aspiration and biopsy (Figure 3):

• Abnormal/lambda monoclonal plasma cell population (5%–7%)
• Kappa/lambda ratio: 0.053
• Amyloid deposition in periadipose tissue and vascular structures confirmed by Congo red staining
• No definitive evidence of plasma cell myeloma

**Figure 3:**
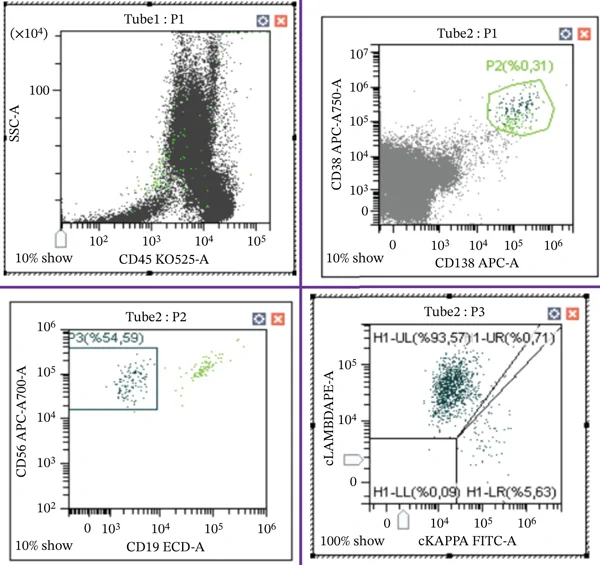
Flow cytometry evaluation.

Urine immunofixation electrophoresis (Figure 4):

• Kappa: 18.4 mg/L
• Lambda: 39.5 mg/L
• Kappa/lambda ratio: 0.47
• Presence of a monoclonal peak

**Figure 4:**
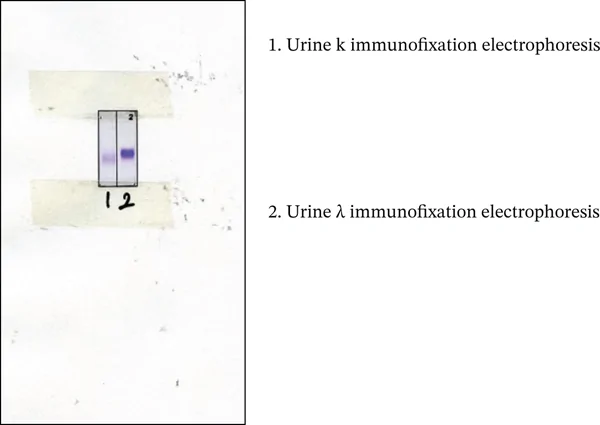
Urine immunofixation electrophoresis.

### 2.2. Imaging Findings

Transthoracic echocardiography:

• Left ventricular ejection fraction: 45%–50%
• Systolic and diastolic dysfunction
• Severe mitral and tricuspid regurgitation
• Minimal pericardial effusion
• Concentric left ventricular hypertrophy
o. These findings were consistent with cardiac amyloidosis.

PET/CT imaging showed no evidence of multiple myeloma or malignancy.

### 2.3. Clinical Course

The patient was started on a chemotherapy regimen including bortezomib, cyclophosphamide, and dexamethasone on June 27th, 2025. Two days later, a syncope attack developed and cardiac and neurological evaluations did not reveal new pathology. However, laboratory findings were consistent with acute kidney injury superimposed on chronic kidney disease (urea: 239 mg/dL; creatinine: 2.76 mg/dL).

On the third hospital day, the patient developed acute atrial fibrillation followed by asystole. Spontaneous circulation was restored after a brief cardiopulmonary resuscitation. Following stabilization, an implantable cardioverter defibrillator (ICD) was placed to patient in the Coronary Intensive Care Unit (ICU) on July 22nd. The patient’s clinical condition improved, and a second cycle of chemotherapy was administered on July 24th in the general ward.

### 2.4. Intensive Care Course and Assessment at ICU Admission

Following the second treatment cycle, the patient developed massive ascites necessitating two paracentesis procedures. Blood cultures obtained because of a febrile episode on August 8—1 day following the completion of the second chemotherapy cycle—yielded *Pseudomonas* species, and the patient was transferred to the ICU on August 14th due to the onset of sepsis and septic shock. The patient’s physical examination on admission to the ICU was as follows:

• Mental status: Confused (GCS: 13)
• SOFA score: 12
• APACHE II score: 25
• Abdominal: massive ascites, pleural and pericardial effusion
• Periorbital: typical amyloid purpura on the left eyelid (Figure 5)
• Oliguria and severe metabolic acidosis

**Figure 5:**
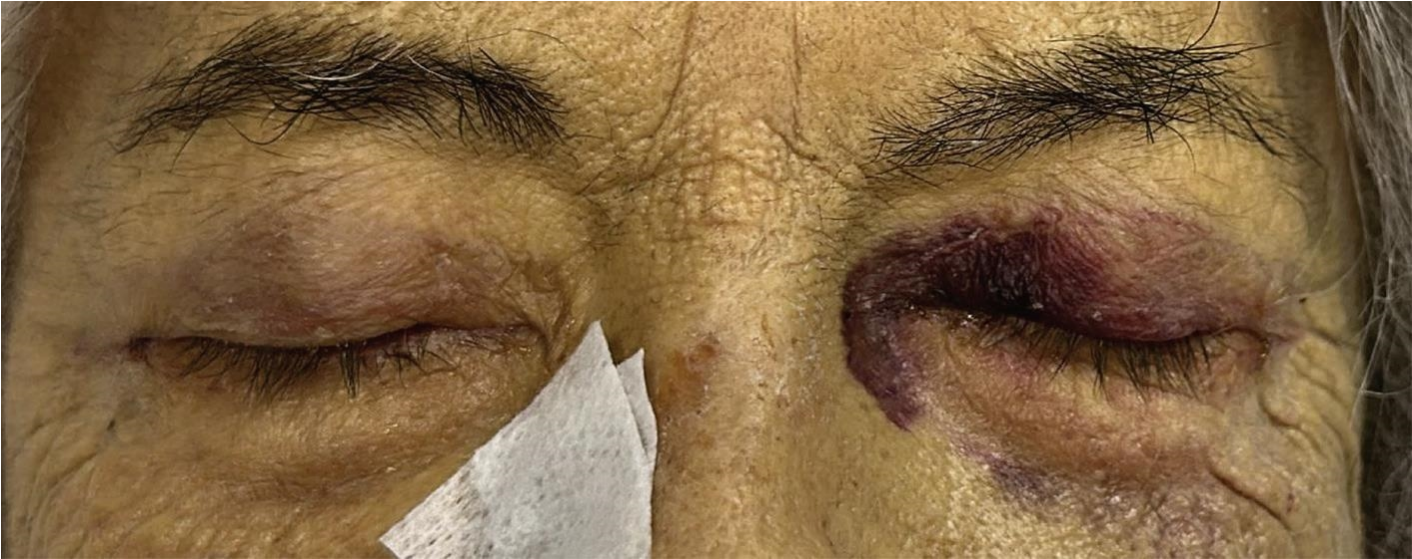
Ocular amyloidosis (Amyloid purpura).

The patient’s laboratory parameters at the ICU admission have been given in Table 1. And C‐reactive protein, procalcitonin, and NT‐ProBNP courses have been shown in Figures 6, 7, and 8.

**Table 1:** The patient’s laboratory parameters at the ICU admission.

Laboratory parameter	Patient values	Units	Reference values
Urea	308	mg/dL	12.5–42.8
Creatinine	3.04	mg/dL	0.55–1.02
Alanine aminotransferase	97	IU/L	7–40
Aspartate aminostransferase	178	IU/L	< 40
Alcaline phosphatase	114	IU/L	34–107
Lactate dehydrogenase	711	IU/L	120–246
Total bilirubin	1.4	mg/dL	0.2–1.2
C‐reactive protein	4.9	mg/dL	< 0.5
Procalcitonin	2.3	*μ*g/L	< 0.1
Platelet count	36,000	Per *μ*L	150,000–439,000
Leukocytes	12,160	Per *μ*L	4,060–10,600
Neutrophil‐to‐lymphocyte ratio	20.8		< 5.5
Lactate level	2.5	mmol/L	< 1.5
NT‐ProBNP	45,709	ng/L	< 300

**Figure 6:**
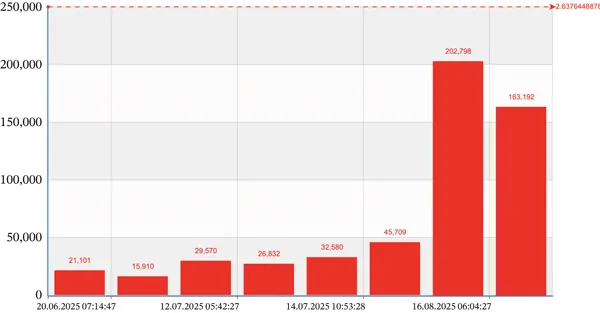
The patient’s NT‐proBNP values in our hospital (ng/L).

**Figure 7:**
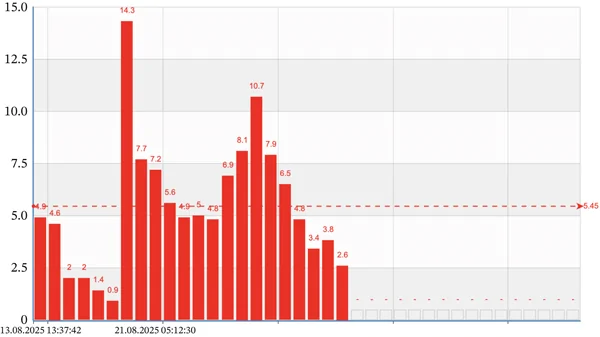
The patient’s CRP values at the time of ICU admission, Aug 14th (mg/dL).

**Figure 8:**
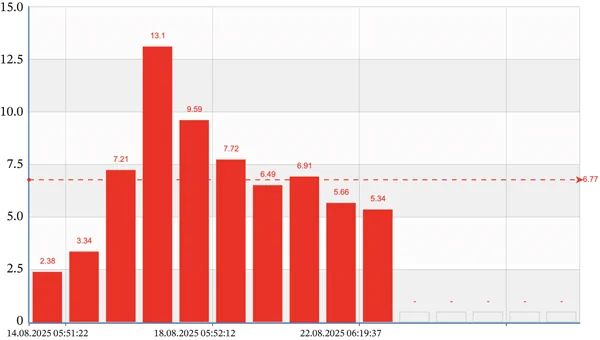
The patient’s procalcitonin values at the time of ICU admission (*μ*g/L).

#### 2.4.1. Culture Results

• *Pseudomonas guariconensis* (port and peripheral blood; susceptible to meropenem, colistin, amikacin and ceftolozane/tazobactam and resistant to aztreonam, cefepime, ceftazidime, ciprofloxacin, imipenem, levofloxacin, piperacillin‐tazobactam)
• *Klebsiella pneumoniae* (urine; susceptible to meropenem and amikacin only)
• *Stenotrophomonas maltophilia* (rectal swab)

#### 2.4.2. Antimicrobial Therapy

• Meropenem, fluconazole (both started on August 8th)
• Linezolid and polymyxin B were subsequently added on August 15th due to the deterioration of the patient’s clinical condition.

During the ICU stay, the patient required mechanical ventilation and hemodynamic support, including a continuous infusion of noradrenaline. Suspecting the venous port as the source of the *Pseudomonas guariconensis* bacteremia, the port was promptly removed, and a temporary internal jugular venous catheter was placed for continued vascular access. A catheter was placed for ascitic fluid drainage, and a pleurocan was inserted to manage a right‐sided pleural effusion. Renal replacement therapy was not required during the ICU stay; the acute kidney injury was successfully managed with conservative measures and subsequently resolved. Following improvement in respiratory function and the discontinuation of vasopressor therapy, the patient progressed to the point of extubation. Once her clinical condition stabilized, she was transferred to the general ward for continued care on September 3rd (Table 2).

**Table 2:** Timeline of clinical course and interventions.

Date/timeframe	Clinical milestone	Key findings and interventions
5 months Prior	Initial presentation and diagnosis	Symptoms: Edema, dyspnea, weight loss. Diagnosed with cardiac AL amyloidosis.
June 27, 2025	1st chemotherapy cycle	Bortezomib, cyclophosphamide, and dexamethasone initiated.
June 29, 2025	Clinical deterioration	Syncope attack; acute kidney injury developed.
June 30, 2025	Cardiac arrest	Atrial fibrillation progressing to asystole; successful CPR.
July 22, 2025	Device implantation	Implantable cardioverter‐defibrillator (ICD) placed.
July 24, 2025	2nd chemotherapy cycle	Second cycle started; complicated by massive ascites requiring paracentesis.
August 8, 2025	Febrile episode and cultures drawn	Febrile episode 1 day after completing the 2nd cycle. Blood cultures were drawn. Empiric meropenem and fluconazole started.
August 14, 2025	Septic shock and ICU admission	Clinical decline 7 days post‐chemotherapy. Admitted to ICU (SOFA: 12, APACHE II: 25). Required mechanical ventilation and noradrenaline.
August 15, 2025	Pathogen identification and source control	Blood cultures drawn on August 8 yielded *Pseudomonas guariconensis*. Port removed; jugular catheter placed. Linezolid and polymyxin B added.
September 3, 2025	Recovery and ICU discharge	Extubated, vasopressors discontinued; transferred to the general ward.

## 3. Discussion

This case illustrates a presentation of AL amyloidosis with prominent cardiac and renal involvement, rapid deterioration related to infection and septic shock.

The consensus group recommends defining sepsis‐3 as a condition characterized by life‐threatening organ dysfunction, identified by an increase of at least 2 points in the SOFA subscore for the affected organ, arising from a dysregulated host response to infection [5]. The superimposition of infection on preexisting organ dysfunctions has been also defined as sepsis. The presence of infection may not always be clinically apparent; thus, identifying sepsis solely by evaluating organ dysfunction in a patient with a confirmed infection is not always feasible. More commonly, clinicians adopt the inverse approach, considering whether the acute development of unexplained hypoxemia, oliguria, thrombocytopenia, or altered mental status may indicate the onset of sepsis in their patient [4]. An additional critical aspect is the early identification of sepsis. While the SOFA score is widely acknowledged as a reliable tool for assessing organ dysfunction, its complexity limits its practicality for early sepsis detection outside the ICU. Notably, the SOFA score reflects both chronic and acute alterations in organ function, Quick SOFA (qSOFA) may help the clinicians at these situations [6].

Although the early diagnosis rate of amyloidosis remains relatively low, the prevalence of the amyloidosis increases with age; specifically, it was twice as high in individuals aged 65 and older compared with those between 35 and 54 years [7]. In patients over 40, typical presentations may include nephrotic syndrome with low blood pressure, hepatosplenomegaly, and multisystem involvement. By highlighting the multisystemic nature of the disease process, we seek to contribute to the existing body of knowledge, thereby promoting earlier recognition of infection with existing organ dysfunctions and facilitating prompt and effective therapeutic intervention. Early recognition, accurate staging, and prompt initiation of therapy are essential determinants of prognosis of amyloidosis [8]. Clinical suspicion should be raised in adults with the following features: nondiabetic nephrotic syndrome; heart failure with preserved ejection fraction, particularly with restrictive physiology; unexplained hepatomegaly, distal sensory neuropathy; or atypical monoclonal gammopathy [9]. Laboratory tests often reveal monoclonal immunoglobulin. Notably, even in cases of end‐stage renal disease, kidney volume typically remains unchanged, which may indicate the presence of amyloid nephropathy [10]. The kidneys are among the organs most commonly affected in amyloidosis. Because of the lack of specific clinical symptoms, AL renal amyloidosis is often misdiagnosed or overlooked in clinical practice. Definitive diagnosis typically requires a renal biopsy. Consistent with Gertz and Dispenzieri’s systematic review, early cardiac involvement in AL subtype is a critical determinant of prognosis [3]. The reported short‐term mortality for advanced cardiac AL amyloidosis is significantly high, especially in patients with NT‐proBNP elevation, low EF, and arrhythmia [2]. Our patient had NT‐proBNP values over 200,000 ng/L during the ICU period.

AL‐type amyloidosis is fundamentally considered a complication of plasma cell dyscrasia, and its treatment strategies are largely adapted from multiple myeloma chemotherapy protocols. These therapies aim to rapidly eliminate circulating light chains by specifically targeting plasma cells, thereby reducing the production of amyloid precursor proteins [11].

In our case, monoclonal light chain (lambda) was confirmed via multiple assays and despite no myeloma, plasma cell dyscrasia with light chain overproduction led to systemic organ depositions and organ dysfunctions. During the treatment period with bortezomib‐based chemotherapy, infection and sepsis have been developed due to immunosuppression. Systemic AL amyloidosis remains a diagnostic challenge due to nonspecific symptoms. Our patient, previously healthy, progressed rapidly to multi‐organ involvement, emphasizing the need for early suspicion, multidisciplinary care, and prompt treatment initiation. Despite therapy, cardiac involvement and sepsis significantly impacted prognosis.

As a conclusion, there is clear consensus on key aspects of sepsis, including the critical role of organ dysfunction and the imperative for early recognition. This clarity is expected to facilitate a more standardized approach to the diagnosis and management of the septic patient with amyloidosis.

This study is limited by its retrospective, single‐case design, which precludes generalizability, and the extreme rarity of *Pseudomonas guariconensis*, restricting extensive literature comparisons. Future prospective studies and registries are needed to optimize antimicrobial strategies and clarify immune dysregulation in AL amyloidosis‐associated sepsis. Further investigation into the ‘two‐hit’ hypothesis (sterile inflammation followed by infection) may ultimately guide targeted immunomodulatory interventions for this vulnerable population.

### 3.1. Learning Points

The superimposition of infection on preexisting organ dysfunctions has been also defined as sepsis.

Systemic amyloidosis should be considered in patients who present with nondiabetic proteinuria, heart failure with preserved ejection fraction, unexplained peripheral neuropathy, or atypical manifestations of monoclonal gammopathy.

Delays in the diagnosis of systemic amyloidosis, frequently resulting from unrecognized clinical presentations, can adversely affect patient outcomes.

Early detection of AL amyloidosis is crucial, as prompt initiation of chemotherapy has the potential to markedly enhance organ function and improve overall prognosis.

During the hospitalization period of patients with amiloidosis related to organ dysfunctions, earlier recognition of infection and septic shock reduces mortality.

## Funding

No funding was received for this manuscript.

## Conflicts of Interest

The authors declare no conflicts of interest.

## Supporting information


**Supporting Information** Additional supporting information can be found online in the Supporting Information section.  

## Data Availability

The data that support the findings of this study are available from the corresponding author upon reasonable request.
